# Sensitivity of influenza virus to ultraviolet irradiation

**DOI:** 10.3205/dgkh000423

**Published:** 2022-10-26

**Authors:** Martin Hessling, Anna-Maria Gierke, Ben Sicks, Nicole Fehler, Petra Vatter

**Affiliations:** 1 Institute of Medical Engineering and Mechatronics, Ulm University of Applied Sciences, Ulm, Germany

**Keywords:** influenza virus, virus type, disinfection, inactivation, ultraviolet, UVA, UVB, UVC, far-UVC

## Abstract

**Background:**

The measures implemented against the coronavirus pandemic also led to a sharp decline in influenza infections in the 2020/2021 flu season. In the meantime, however, the number of influenza infections has risen again; it is known from history that influenza viruses can also trigger severe pandemics. Therefore, we investigated the efficacy of ultraviolet radiation in the spectral range of 200–400 nm for inactivating influenza viruses.

**Materials and methods:**

The scientific literature was searched for published ultraviolet (UV) irradiation experiments with influenza viruses and the results were standardized by determining the lg-reduction dose. The results were then sorted and analyzed by virus type and wavelength as far as possible.

**Results:**

The scope of the published data sets was limited and revealed large variations with regard to the lg-reduction dose. Only for experiments with influenza viruses in liquid media in the UVC spectral range around 260 nm – the emission range of commonly-used mercury vapor lamps – was there sufficient data to compare virus types. No significant difference between the virus (sub-) types was observed. The lg-reduction dose in this spectral range is 1.75 mJ/cm^2^ (median). It was also shown that influenza viruses are particularly sensitive in the far-UVC spectral range (200–230 nm).

**Conclusion:**

UVC, including far-UVC, is suited for influenza virus inactivation as long as the viruses are in UVC-transparent materials. A large difference in the UV sensitivity of different influenza viruses from the last approx. 100 years could not be detected. Thus, it is reasonable to assume that future influenza viruses will also be similarly UV-sensitive or that UV can also inactivate new influenza viruses.

## Zusammenfassung

**Hintergrund:**

Die gegen die Coronavirus-Pandemie durchgeführten Maßnahmen haben in der Saison 2020/2021 auch zu einem starken Rückgang von Grippeinfektionen geführt. Mittlerweile ist die Zahl der Grippeinfektionen aber wieder angestiegen und aus der Historie ist bekannt, dass auch Grippeviren Auslöser schwerer Pandemien sein können. In der vorliegenden Arbeit soll daher untersucht werden wie wirksam ultraviolette Strahlung im Spektralbereich 200–400 nm zur Inaktivierung von Grippeviren ist.

**Material und Methoden:**

In der wissenschaftlichen Literatur wurde nach UV-Experimenten mit Grippeviren gesucht und die Resultate vereinheitlicht, indem die lg-Reduktionsdosis bestimmt wurde. Die Ergebnisse wurden, soweit möglich, nach Virus-Typ und Wellenlänge analysiert.

**Ergebnisse:**

Der Umfang der publizierten Datensätze ist begrenzt und weist im Hinblick auf die Reduktionsdosis große Variationen auf. Nur für Experimente mit Grippeviren in flüssigen Medien im UVC-Spektralbereich um 260 nm, in dem auch weit verbreitete Quecksilberdampflampen emittieren, finden sich ausreichend viele Daten, um die Empfindlichkeit unterschiedlicher Virus-Typen zu vergleichen. Ein signifikanter Unterschied zwischen den Virus- (Sub-) Typen ist nicht zu erkennen. Die Reduktionsdosis in diesem Spektralbereich liegt bei 1,75 mJ/cm^2^ (Median). Es zeigt sich außerdem, dass Grippeviren im Far-UVC-Spektralbereich (200–230 nm) besonders empfindlich sind.

**Schlussfolgerung:**

UVC, inklusive Far-UVC, ist prinzipiell zur Inaktivierung von Grippeviren geeignet, solange sich die Viren in UVC-transparenten Materialien befinden. Ein Unterschied in der UV-Empfindlichkeit verschiedener Grippeviren aus den letzten ca. 100 Jahren ist nicht erkennbar. Das gibt Grund zu der Annahme, dass auch zukünftige Grippeviren ähnlich UV-empfindlich sein werden bzw. dass UV-Strahlung auch neue Grippeviren inaktivieren kann.

## Introduction

Prior to 2019, 290,000 to 650,000 people died each year worldwide as a result of influenza infections [1]. With the emergence of the coronavirus pandemic, this number has decreased significantly. The use of personal protective equipment, reduction of social contacts and other factors are assumed to be the cause [2]. In the influenza season 2021/2022, however, the number of influenza infections rose significantly again and already seems to have reached the level of earlier years [3].

Influenza is caused by enveloped, single-stranded RNA viruses from the Orthomyxoviridae family [4], [5]. Influenza viruses are often more or less spherical with diameters between 80 and 120 nm [4]. Among other things, the various genera differ in the number of their RNA segments. Influenza viruses of type A or B contain 8 RNA segments, and types C and D 7 segments. Of particular medical importance are types A and B, which cause seasonal flu in varying proportions. For example, during the last influenza season in 2021/2022, 93.5% of the influenza viruses identified were type A and 6.5% were influenza B [3].

The greatest threat with regard to a pandemic probably comes from influenza A viruses. Historically, this is supported by the fact that all influenza pandemics of the last 100 years were triggered by influenza A viruses. For example, the pathogen that caused the Spanish flu of 1918, which is said to have claimed 50 million lives or more [6], [7], was an influenza A virus of the subtype H1N1. In the Asian flu pandemic of 1957 and the Hong Kong flu pandemic of 1968, the influenza A viruses were H2N2 and H3N2 [8]. H1 through H18 and N1 through N11 are different viral hemagglutinin and neuraminidase surface proteins that the virus needs to infect the host cell and to multiply. 

From a biological point of view, there are also reasons why influenza A viruses pose a particular danger. The genome is not stable, but evolves constantly through antigenic drift and antigenic shift, making immune defense more difficult. This genetic variability also allows them to jump across species boundaries. Waterfowl are considered to be the natural reservoir of influenza A viruses. These influenza viruses can either cause infections in humans directly or via intermediate hosts, such as pigs. For example, it is suspected that the Spanish flu of 1918 was also transmitted from pigs to humans [8].

Potter expects influenza pandemics every 10–40 years [9], but there is no “fixed schedule”. Theoretically, for instance, an influenza A virus from the mentioned influenza reservoir of waterfowl can also lead to an infection in humans at any time. If this is a new influenza A virus, for which there is still no immunity in the population, it can develop into a pandemic.

Similar to the coronavirus pandemic, measures will then be necessary to help inactivate the virus or contain its spread. The disinfection techniques known so far, such as chemical disinfectants, thermal disinfection or ultraviolet (UV) radiation, will probably also help against a possible new flu virus.

This literature review is about the application of ultraviolet radiation especially at the emission wavelength of conventional mercury vapor lamps. Of particular interest is also the far-UVC range (200–230 nm), which promises a strong antimicrobial and virucidal effect with low risk to humans [10], [11], [12], [13]. The intention is to provide estimates for the lg-reduction doses for future influenza viruses. For this purpose, previously available UV disinfection data for already known influenza viruses will be examined for their UV sensitivity – given as lg-reduction doses – in different media in order to reveal similarities and differences.

## Materials and methods

Pubmed and Google Scholar were searched for various combinations of the following terms: “influenza”, “flu”, “disinfection”, “inactivation”, “reduction”, “radiation”, “irradiation”, “UV”, “ultraviolet”, “UVC”, “UV-C”, “UVB”, “UV-B”, “UVA” and “UV-A”. The references in the literature found were examined to see if they could be included in the study. We also checked whether the retrieved studies were cited by other relevant literature. The data collected was not limited to human influenza viruses; information on animal influenza viruses was also evaluated.

The paramount property investigated in this study was the sensitivity of the viruses to UV radiation, expressed as the lg-reduction dose needed for a 90% virus inactivation. If this information was not explicitly given in the text or in tables, an attempt was made to determine the lg-reduction dose from graphs. These lg-reduction doses were calculated under the assumption of exponential behavior, as observed by several authors [14], [15], [16], [17], [18], [19], [20], [21].

It is known that the entire UV spectral range has an antimicrobial effect, but also that large differences of more than a factor of 1,000 can exist between different UV sub-ranges. Therefore, the results found were grouped according to wavelength ranges: [200–230 nm] (far-UVC), [231–250 nm], [251–270 nm], [271–290 nm], [291–315 nm], and [316–400 nm]. 

In this way, the different results for the different viruses do not scatter excessively based on the different wavelengths, and in the range of maximum RNA absorption around 260 nm, greater amounts of data can be compared directly to each other. However, due to different experimental setups and biological variations, there is still some scattering even within these intervals, and therefore the main result per interval is the median, which is less sensitive to outliers compared to the average.

## Results

A total of 39 publications on influenza virus inactivation with UV radiation were retrieved. Some of them over 50 years old and contain less precise virus designations than is common today. For example, articles older than 50 years often refer to “the Mel strain” or “the WSN strain”. Searches of other older and more recent sources, particularly the Influenza Research Data Base (https://www.fludb.org/brc/home.spg?decorator=influenza), suggest that these strains are likely H1N1 A/Melbourne/35 and H1N1 A/WSN/33. However, assignment to current designations has not been successful for all influenza virus strains. 

Several studies were not quantitatively evaluable, because irradiation intensity and dose were missing or given in units such as energy per volume, which is not convertible to energy per area without additional information. The irradiation wavelength was also absent several times. If no specific UV wavelength was named by the authors, a low-pressure mercury vapor lamp with a peak emission at 254 nm was assumed, as these have served as the standard antimicrobial UV radiation sources for decades.

Most retrieved results are given in Table 1 (Tab. 1), which lists virus (sub-) type, irradiation wavelength, lg-reduction dose, and sample medium. Exceptions were made for publications on UV irradiation of FFP (filtering facepiece) materials or masks. In such cases, only mean values from each publication were listed and not all published individual results for each filter, as these results depend predominantly on (unknown) UV absorption properties of the filter materials and the number of their layers. This only allows very limited conclusions to be drawn about inactivation properties of influenza viruses.

Most experiments in Table 1 (Tab. 1) were performed in liquid virus suspensions. Only 5 reports on virus irradiation in aerosol could be retrieved, and only for three of them was a quantification of the lg-reduction dose given or possible to be determined. In addition, three results of virus irradiation on surfaces and 5 studies on (FFP) filter materials were available. 

Since mercury vapor lamps with their emission peak at 254 nm have been applied for decades, most published experiments were also conducted at this wavelength or in the spectral range from 251 to 270 nm. The summarized values for all influenza viruses together in this spectral range depending on the sample medium are presented in Table 2 (Tab. 2). There is no significant difference between lg-reduction doses for influenza viruses in liquids and aerosols (p>0.05), but a large difference compared to surface and FFP results. 

To find out whether different influenza (sub-) types exhibit different UV sensitivities, (sub-) types with at least three determined doses in liquids and in the spectral range 251–270 nm were grouped and analyzed. H5N1 and H5N2 subtypes had to be merged to obtain three doses. No influenza B virus was included, as there is only a single dose. 

The median doses for reduction of influenza A virus subtypes H1N1, H3N2, H5N1/H5N2 and all influenza viruses by 1 lg are given in Table 3 (Tab. 3). ANOVA revealed that the doses for these subtypes in liquids are not significantly different from each other (p>0.05). 

The wavelength dependence of the dose for all influenza viruses in liquids is summarized in Table 4 (Tab. 4). In the most important spectral range around 260 nm, the median dose is 1.75 mJ/cm^2^ and in the far-UVC range it is 0.48 mJ/cm^2^. For longer UV wavelengths, the doses increase by several orders of magnitude.

## Discussion

The number of quantitative UV photoinactivation publications is rather small, given the importance of influenza and the number of different virus types and subtypes. For example, there is only one evaluable result for one influenza B strain, and it was published 40 years ago. Nevertheless, UV radiation turns out to be antivirally active against all influenza viruses; large differences in sensitivity between the different virus types or subtypes are not evident. The necessary lg-reduction for the influenza B strain at 254 nm is somewhat lower than for the median for the influenza A viruses. However, as mentioned above, it is based on a single result and still within the range of the influenza A results. This is not surprising, as the RNA strand lengths of about 13.4 and 14.4 kb [22] for influenza A and B subtypes, respectively, are UVC targets of similar size. 

The UV dose for reduction by 1 lg in the most important spectral range of 251–270 nm, which includes emission from conventional germicidal mercury vapor lamps, is 1.75 mJ/cm^2^ (median). This dose is comparable to the analogous value for coronavirus, which is also a single-stranded enveloped RNA virus [23]. Surprisingly, the spectral response in Table 4 (Tab. 4) does not match RNA absorption, as observed by other authors in experiments with different wavelengths in the same setup [16], [18], [24], [25]. It would have been expected that UVC radiation would be most effective around 260 nm and that the effect would initially decrease somewhat around 240 nm but increase again in the far-UVC around 222 nm. The reason for the observed deviation may be the low number of individual results for the short wavelength intervals, with relatively high statistical scattering. On the other hand, the increased dose with an approximately 10,000-fold increase in wavelength of UVA radiation was to be expected. 

However, it is necessary for UV radiation to actually reach the viruses. Viruses in or behind UV-absorbing materials may not be inactivated or may be inactivated poorly. These properties of UV radiation are sometimes misjudged, when even scientists assume that UVC radiation passes through normal glass, for instance. One should not be deceived by “UV-transparent” designations of plastics and glasses. These may be transparent to UVA radiation (315–400 nm), but usually not to UVC (200–280 nm). The only UVC transparent glass material is fused silica, and even in this case, significant absorption may occur, especially in the far-UVC range (200–230 nm). 

Also, a possible source of error, falsely leading to higher lg-reduction data, is the use of absorbing culture media, such as DMEM. Virus solutions often consist of viruses in such culture media diluted in salt solutions; but even diluted, media absorption can attenuate UVC radiation. This is especially true below 240 nm, when protein absorption increases sharply, and it is also true for virus solutions that appear completely transparent to the human eye.

A similar problem ensues upon irradiation of virus samples in micro-titer plates with many small wells. In wells that are not directly under the UVC radiation source, the well walls shadow part of the wells’ contents, thus reducing disinfection effectiveness. Shadows and absorption may both lead to deviations from mono-exponential virus reduction. 

Conversely, effects also occur that can lead to an erroneously low reduction dose. For example, when metallic sample vessels are used, and part of the radiation is reflected and passes through the sample a second time.

The extent to which the data determined in Table 1 (Tab. 1) (for liquids) depend on non-optimal experimental conditions, or are biological variations, is difficult to establish retrospectively. In general, however, the required UV irradiation doses for influenza viruses in liquids and aerosols are very low. 

It should be emphasized that one lg-reduction dose only leads to 90% virus inactivation and virucidal measures officially require at least 99.99% (4 lg-level) reduction [26], [27]. With the assumption of exponential inactivation behavior, this leads to an assumed 4-fold higher necessary irradiation dose of 7 mJ/cm^2^. With typical recommendations such as 40 mJ/cm^2^ UVC from the drinking water sector [28], influenza virus reduction by many orders of magnitude is possible.

However, the situation on surfaces and in materials such as FFP filters is more difficult. With surfaces, although the reflectivity of the material plays a role, the porosity exerts a greater influence, since deeper pores in particular can provide shade and shield the virus from the radiation.

The statements on FFP filters in Table 1 (Tab. 1) seem relatively consistent, but this is due to the fact that although the relevant studies were conducted with different materials from different manufacturers, only the average disinfection success rates are presented here. In the studies themselves, major unknown differences in detail exist between the products of the different manufacturers and each additional UV-absorbing filter layer, which can substantially influence the disinfection effect. The above-mentioned drinking water standard of 40 mJ/cm^2^ would have to be increased by more than one order of magnitude in this case.

UVB and UVA radiation also exhibit antiviral properties, but the effect is many orders of magnitude weaker than for UVC. Under realistic irradiation conditions, UVB and UVA would require irradiation durations of hours or days to achieve a reduction by several lg-levels.

## Conclusion

UVC, including far-UVC, is suitable for the inactivation of influenza viruses in UVC-transparent liquids and aerosols. Since only very small irradiation doses are required for 90% inactivation, reductions of several orders of magnitude can be achieved within seconds or minutes.

The differences in UV sensitivity of influenza viruses that have emerged over the last 100 years are rather small. UVC radiation has been applied as an antimicrobial measure for more than 100 years, but no concomitant change in the UV sensitivity of influenza viruses has been observed. Therefore, our expectation is that possible future influenza viruses will be similarly UV sensitive or that UVC radiation will be an effective measure against new influenza viruses. 

## Notes

### Competing interests

The authors declare that they have no competing interests.

### Acknowledgment

The authors did not receive any funds.

## Figures and Tables

**Table 1:**
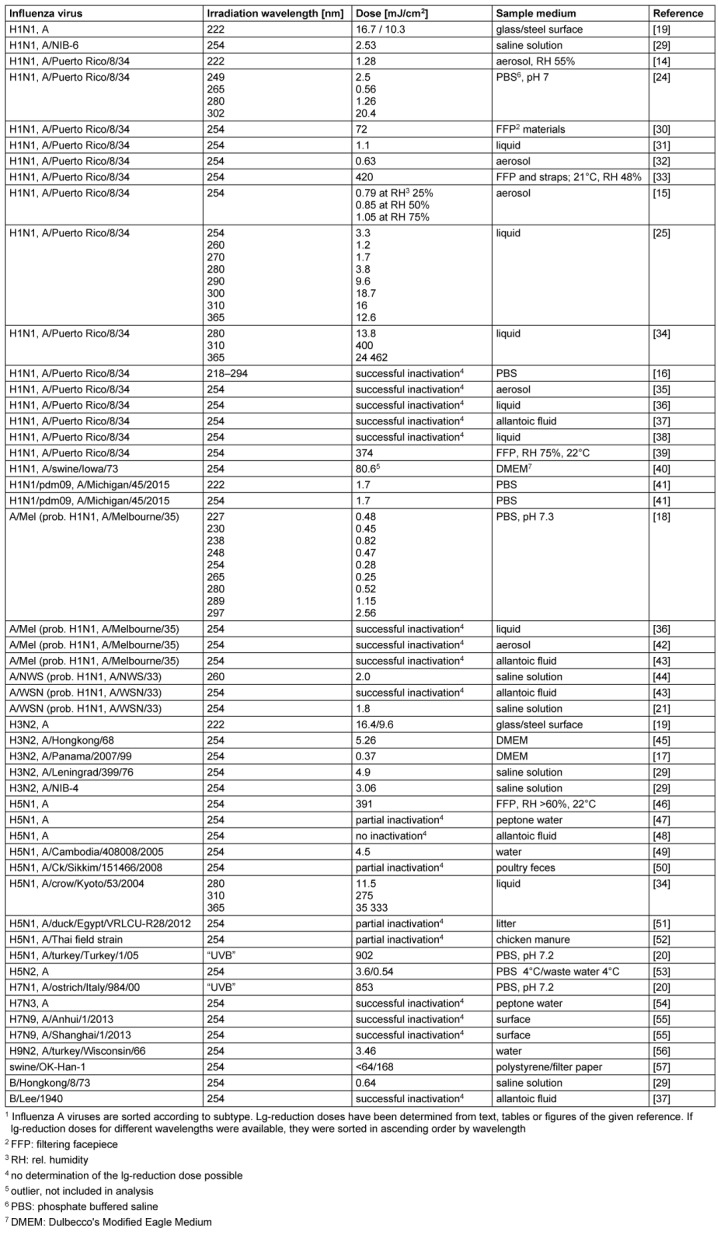
Published ultraviolet irradiation experiments on influenza viruses and determined dosages for 1 lg-reduction

**Table 2:**
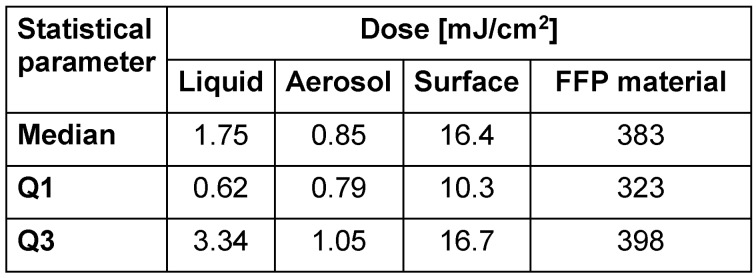
Median dosages, 25%, and 75% quartiles (Q1 and Q3) for reduction of all influenza viruses by 1 lg according to sample medium (liquid, aerosol, surface, and FFP material) in the UV range 251–270 nm

**Table 3:**
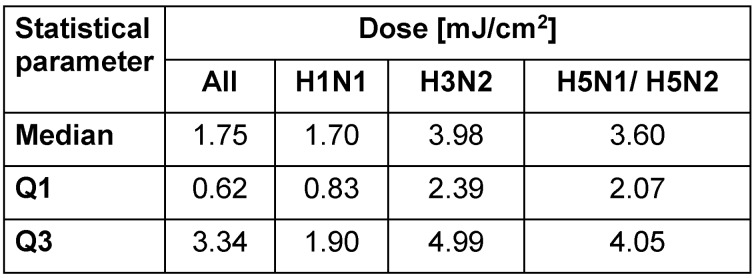
Median dosage, 25%, and 75% quartile (Q1 and Q3) for reduction of different influenza A virus subtypes by 1 lg in liquids in the UV range 251–270 nm

**Table 4:**
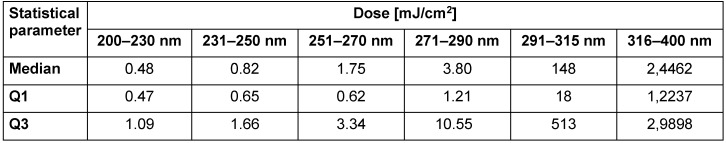
Median dosage, 25%, and 75% quartile (Q1 and Q3) for reduction of all influenza viruses by 1 lg in liquids for different UV intervals
